# Monogenic Lupus: Insights into Disease Pathogenesis and Therapeutic Opportunities

**DOI:** 10.1097/BOR.0000000000001008

**Published:** 2024-02-29

**Authors:** Yuting Qin, Jianyang Ma, Carola G. Vinuesa

**Affiliations:** 1 China Australia Centre for Personalized Immunology (CACPI), Renji Hospital, Shanghai Jiao Tong University School of Medicine (SJTUSM), Shanghai, China; 2 The Francis Crick Institute, London, UK

**Keywords:** Systemic lupus erythematosus, monogenic lupus, complement deficiency, type I interferon, immunotolerance

## Abstract

**Purpose of review:**

This review aims to provide an overview of the genes and molecular pathways involved in monogenic lupus, the implications for genome diagnosis, and the potential therapies targeting these molecular mechanisms.

**Recent findings:**

To date, more than 30 genes have been identified as contributors to monogenic lupus. These genes are primarily related to complement deficiency, activation of the type I interferon (IFN) pathway, disruption of B cell and T cell tolerance and metabolic pathways, which reveal the multifaceted nature of systemic lupus erythematosus (SLE) pathogenesis.

**Summary:**

In–depth study of the causes of monogenic lupus can provide valuable insights into of pathogenic mechanisms of SLE, facilitate the identification of effective biomarkers, and aid in developing therapeutic strategies.

## Introduction

SLE is a systemic autoimmune disease that predominantly affects women, impacting multiple organs such as the kidneys, skin, joints, lungs, cardiovascular system, central nervous system, and hematopoietic system (1). Although the etiology of SLE remains to be fully elucidated, there is increasing realization that it is a disorder of increased nucleic acid sensing or signaling, with genetic and environmental factors contributing to its onset (1). A significant genetic component is underscored by twin studies showing higher concordance rates in monozygotic twins compared to dizygotic twins and familial aggregation (2, 3).

SLE is primarily a polygenic disease, with numerous risk variants identified through genome-wide association studies (GWAS) and an increasing number of functional rare variants identified through whole exome sequencing (WES) and whole genome sequencing (WGS) studies (4). Variants identified by GWAS are common in the general population, and although most have small or modest effects, they can act additively to increase the risk of developing SLE (5). However, risk alleles identified by GWAS still account for only a small fraction of the genetic risk associated with disease (6). It is increasingly acknowledged that additional more rare variants may contribute to the missing heritability and exert strong effects. These rare variants can occur in GWAS SLE risk genes, or in genes known to cause monogenic lupus, or in genes not yet associated with SLE (7). In recent years, the increasing accessibility and affordability of next-generation sequencing technologies such as WES and WGS have enabled the discovery of novel and rare variants, expanding the list of genes causing monogenic SLE (4). Monogenic lupus, associated with alterations in single genes, is a rare yet severe form of SLE that typically manifests during childhood (8). Despite constituting a small fraction of lupus cases, monogenic lupus greatly enhances our understanding of disease pathogenesis and illuminates therapeutic targets. This article aims to review our current knowledge of the genes involved in monogenic lupus and their corresponding molecular pathways. It outlines a growing role of genomic diagnosis and highlights potential targeted therapies derived from understanding the molecular mechanisms.

### Genetic basis of monogenic lupus

**Figure 1:**
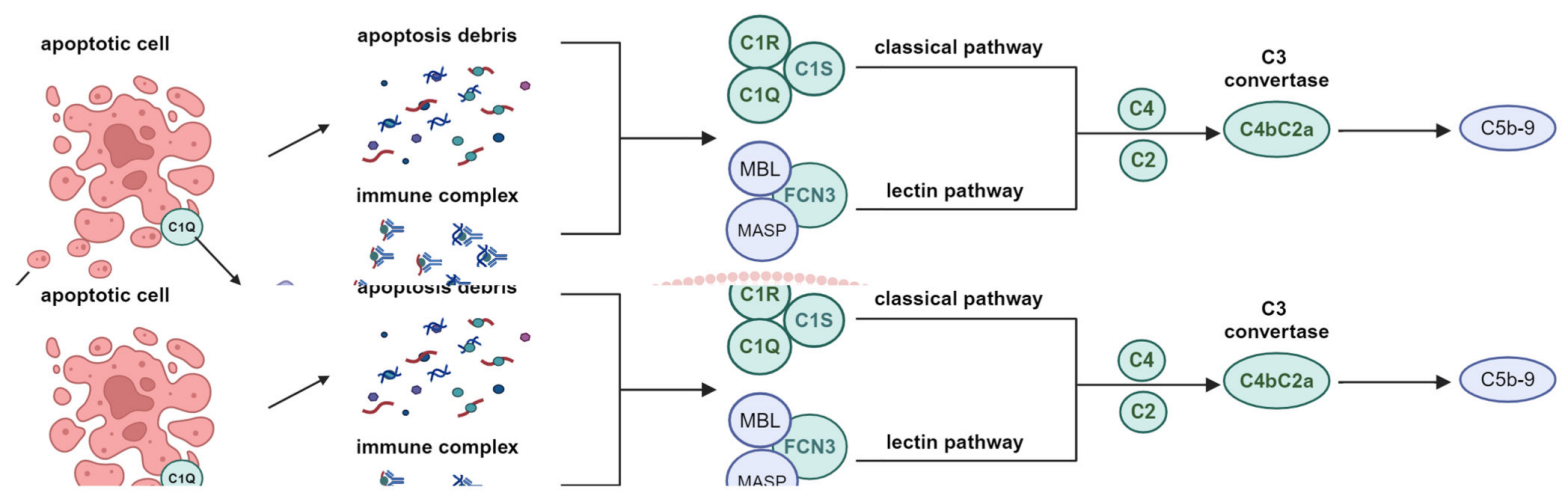
Signaling pathways involved in the pathogenesis of monogenic lupus. (*Created with BioRender.com*) Complement activation, nucleic acid degradation, nucleic acid sensing and signaling, as well as type I IFN signaling, are the main molecular pathways involved in the development of monogenic lupus. LoF variants in genes encoding proteins related to complement activation (including C1Q, C1R/C1S, C2, C4, and FCN3) and extracellular nucleic acid degradation (DNASE1, DNASE1L3, and SAT1) prevent clearance of apoptotic cellular debris, mitochondrial DNA, and neutrophil extracellular traps. Accumulation of nucleic acids stimulates the type I IFN pathway. This pathway can also be overactivated by LoF variants in genes encoding proteins linked to intracellular nucleic acid sensing and/or degradation (including *DNASE2, TREX1, SAMHD1, RNASEH2, c-GAS/*STING, RIG-I, and MDA5). The endosomal ssRNA receptor TLR7 can also lead to SLE, and TLR7 signaling can be restrained by TLR9. Variants in genes that regulate TLR7/MyD88 and/or type I IFN signaling (such as ACP5, PACSIN1, IKAROS, A20, ISG15, UNC93B1 and NLRC4) have also been shown to cause SLE. Proteins in red indicate GoF (Gain of Function); green indicate LoF (Loss of Function). Dashed lines: mechanism is still uncertain.

The pathogenesis of SLE is complex, encompassing abnormalities across all facets of the immune system. Central to SLE pathogenesis is a breakdown in B cell tolerance to nucleic acids and activation of the type I IFN pathway and complement. Most gene variants implicated in monogenic lupus relate to these interconnected processes. (Table 1 & Figure 1).

#### Complement deficiency

The complement system, consisting of a complex network of proteins and receptors, is crucial for host defense against pathogens and maintenance of homeostasis by removing immune complexes and cell debris. It can be activated through various triggers and operates mainly via three pathways: the classical, lectin, and alternative pathway. Each pathway concludes with the creation of C3 convertases and shares a common final step leading to the assembly of the membrane-attack complex (MAC), which creates a pore in the target cell, promoting osmotic cell lysis.

Homozygous deficiencies or loss-of-function (LoF) variants in the early components of the classical pathway were the first identified causes of monogenic SLE (9). This pathway begins with C1q binding to immune complexes, sequentially activating C1R and C1S to form the C1 complex. Once this complex is formed, C1S activates C4 and C2, resulting in the formation of the C3 convertase (also known as C4b2a) (10). The incidence rate of SLE or SLE-like phenotypes in the presence of *C1Q*, *C1R/C1S*, *C4*, and *C2* deficiencies is approximately 90%, 65%, 75%, and 10% respectively (11). In addition, another complement protein in the lectin pathway, FICOLIN-3 (encoded by *FCN3*), has also been linked to SLE. *FCN3* deficiency can increase the risk of developing SLE, with an incidence rate of around 30% (12, 13).

At least three mechanisms have been proposed to explain how deficiency in complement components causes SLE. The first one relates to ineffective clearance of apoptotic debris, which exposes nucleic acid autoantigens and increases their burden. Indeed, C1Q, C1R, C1S, C2, C3 and C4 can mediate opsonization of apoptotic cells. C1Q can also directly bind to apoptotic cells and ligate calreticulin and CD91 on phagocytes, thus enabling phagocytosis (14). A second mechanism involves negative regulation of type I IFN production by C1Q. This is achieved by C1Q preferentially promoting the binding of immune complexes to monocytes and thus preventing these complexes from attaching to, and activating, plasmacytoid dendritic cells (pDCs), which are a major source of type I IFN (15). The third mechanism involves the role of complement receptors 1 (CR1/CD35) and 2 (CR2/CD21) in maintaining immune tolerance through B cell intrinsic or extrinsic mechanisms. In the hen egg lysozyme (HEL) model, CR1/CR2 or C4 deficient self-reactive B cells fail to undergo anergy (16). One possible explanation is that CD21 can form a co–receptor with CD19 and CD81 on B cells to lower the threshold to BCR-signaling (17). CR1/CR2 are first expressed at the T1-T2 transitional stage in which a lower threshold of BCR signal can increase the sensitivity of the transitional B cell to C4b coated self-antigens and result in cell death (18). Apart from the expression on B cells, CR1/CR2 are highly expressed on follicular dendritic cells (FDCs), and help these cells to bind and present C3d-opsonized auto-antigen, thereby delivering ligands to autoreactive B cells circulating through follicles, which die shortly afterwards in the absence of T cell help (19). More recent studies have suggested additional protective roles of complement in SLE. For instance, C1q has been found to modulate the mitochondrial metabolism of CD8(+) T cells, thus inhibiting autoreactive responses to self-antigens (20).

Despite the overall protective effects of complement with regards to SLE development, detrimental roles of C1Q in SLE have also been suggested: C1Q appears to prevent the degradation of neutrophil extracellular traps (NETs), which can induce type I IFN (21), and mediates microglia-driven destruction of synaptic terminals in neuropsychiatric lupus (22).

#### Activation of the type I IFN pathway

Type I IFN, including IFN–*α* and IFN–*β*, play a crucial role in the protective immune response against viral infections and the regulation of immune cell functions. Activation of type I IFN signaling is a hallmark of SLE (23). Moreover, several monogenic forms of lupus are classified as type I interferonopathies due to their prominent type I IFN signature (24). There are three distinct mechanisms through which the overactivation of type I IFN pathway can occur:

##### Defects in nucleic acid metabolism

Nucleic acid metabolism plays crucial roles in various biological processes, and includes DNA and RNA degradation, DNA replication, DNA repair, and RNA editing. Defects in nucleic acid metabolism lead to the accumulation of self-nucleic acids, which can initiate autoimmunity.

Lupus–relevant nucleic acids can derive from both extracellular and intracellular sources. Extracellular DNA usually generates through processes such as apoptosis, efferocytosis, NETosis, or extrusion of mitochondrial DNA from neutrophils, eosinophils and platelets (23). DNASE1L3 digests double-stranded DNA (dsDNA) derived from chromatin within apoptotic cell microparticles (25). Loss-of-function variants of this enzyme has been reported to cause SLE (26). DNASE1 degrades dsDNA present in NETs (27) but its role in SLE is less clear with a single case reported in 2001 (28).

Intracellular nucleic acids originate from replication stress, DNA or mitochondrial damage, and endogenous retroelement activation (23). Variants in genes involved in DNA or dNTP degradation (*DNASE2*(29–31), *TREX1 and SAMHD1*(32–36)) and RNA degradation (*RNASEH2A*, *RNASEH2B* and *RNASEH2C*(37)) (reviewed in 2018 (38)), are often associated with elevated levels of IFN–α and the emergence of familial chilblain lupus (FCL) (39) and childhood-onset SLE, as well as the classical interferonopathy Aicardi-Goutières syndrome (AGS) (24). Notably, *CECR1*, encoding adenosine deaminase 2 (ADA2), which when defective causes deficiency of adenosine deaminase 2 (DADA2) and functions as an extracellular deaminase (40), has been recently described as a lysosomal DNase and an activator of type I IFNs (41). Interestingly, two reports show several DADA2 patients exhibit manifestations like SLE (42, 43), with one report identifying abnormal activation of type I IFN in two sibling patients (42). Another protein that when deficient causes monogenic SLE is *SAT1* (44). *SAT1* encodes a rate-limiting enzyme in polyamine catabolism. Excessive polyamines may trigger SLE through their ability to bind DNA and induce NETosis; *SAT1* is highly expressed in neutrophils (44).

##### Increased sensitivity, constitutive activation, or increased signaling from nucleic acids sensors

Extracellular and intracellular nucleic acids can be sensed by a set of pattern recognition receptors (PRR) including endosomal TLR7 and TLR9, as well as cytoplasmic STING (encoded by *TMEM173*), RIG-I (encoded by *DDX58*), and MDA5 (encoded by *IFIH1*). Gain-of-function (GoF) variants in *TLR7* (45) and *IFIH1* (46) have been reported to cause monogenic SLE as a result of enhanced sensitivity or constitutive activation, leading to a prominent type I IFN signature. *TLR7* GoF has been shown to cause a B cell–intrinsic breach in tolerance (45) promoting the survival of BCR-activated cells and differentiation into CD11c^+^ age-associated B cells (ABCs) and plasma cells via an extrafollicular pathway. Additionally, there have been two reports with GoF variants in *TMEM173* causing inflammatory syndrome and lupus-like disease (47), as well as FCL (48). Furthermore, a study identified a novel *DDX58* GoF variant in five unrelated families associated with lupus nephritis (49). However, it should be noted that most variants in *TMEM173* (50) and *DDX58* (51) have been reported to cause STING-associated vasculopathy with onset in infancy (SAVI) and Singleton-Merten syndrome (SMS), respectively.

More recently, LoF variants in *UNC93B1,* which is important for endosomal transport and degradation of TLR7, has been reported to cause monogenic lupus (52, 53). Additional causes or contributors to monogenic SLE that are potentially linked with increased nucleic acid sensing include LoF variants in *ACP5* and *PACSIN1*, and GoF variants in *NLRC4*. *ACP5* encodes for tartrate-resistant acid phosphatase (TRAP), which controls the phosphorylation of osteopontin (OPN), a cytokine necessary to produce type I IFN following TLR7/9 and RIG-I like receptors (RLRs) stimulation (54, 55). LoF variants in *ACP5* have been associated with Spondyloenchondrodysplasia (SPENCD), a rare skeletal disorder that often presents with lupus-like characteristics (56). PACSIN1 limits TLR7 signaling through disrupting TRAF4-mediated inhibition of TRAF6 (57). NLRC4, a member of the NOD-like receptor family that regulates inflammasome activation has also been reported to be a positive regulator of TBK1, thereby increasing IFN-β production (58), and causing SLE when overactive (59).

Other negative regulators of TLR/MyD88 signaling have also been shown to be critical for survival of self-reactive B cells and cause SLE when defective. These include *IKZF1* and *TNFAIP3. IKZF1*, encoding the crucial hematopoietic transcription factor IKAROS, has also been linked to SLE, through defective transcription of negative regulators of MyD88 (60, 61). Although *IKZF1* mutations are found in common variable immunodeficiency (CVID), there have been at least two reports of patients with *IKZF1* LoF variants developing SLE (62, 63). *TNFAIP3* encodes the A20 protein, a ubiquitin modifying enzyme that negatively regulates TLR7 signals leading to NF-kB activation (64). A20’s role in limiting B cell survival appears crucial to prevent autoimmunity (65). Three reports of patients with lupus described *TNFAIP3* variants impairing A20-mediated deubiquitination, thought to be causal (66–68).

##### Aberrant type I IFN signaling transduction

Nucleic acid sensors trigger a signaling cascade upon activation involving MYD88/TRAF6 or TBK1 and either IRF7, IRF5, or IRF3, leading to the production of type I IFNs. These interferons bind to receptors IFNAR1 and IFNAR2, subsequently activating the Janus kinase (JAK)-signal transducer and activator of transcription (STAT1/2) signaling pathway, which initiates the expression of IFN-stimulated genes (ISGs) (69). Notably, *TLR7* and *STAT1/2* are also ISGs, establishing a positive feedback loop that enhances nucleic acid sensing and antigen-presenting capacity, as well as supporting plasma cell differentiation and autoantibody production (23).

Several genes encoding proteins involved in the regulation of type I IFN signaling have been linked to SLE. For example, LoF variants in ISG15, which controls the stability of USP18 (70) – a negative regulator for IFN signaling, often cause AGS. However, there is a case report of *ISG15* deficiency causing monogenic lupus (71).

#### Breakdown of B and T cell tolerance

The hallmark of SLE is a loss of B cell tolerance, resulting in the inability to eliminate autoreactive B cells, which go on to differentiate into autoantibody-secreting plasma cells. Loss of T cell tolerance and increased nucleic acid signals can also cause or contribute to a breach in B cell tolerance, as exemplified above with the TLR7 GoF variants.

Monogenic conditions that typically cause autoimmune lymphoproliferative syndrome (ALPS), may present with SLE-like disease (72–77). ALPS can be caused by germline or somatic LoF variants in the *FAS* and *FASLG* genes, and LoF variants in *PRKCD*. FAS and FASL mediate activation-induced cell death, and their deficiency prevents elimination of chronically activated – including autoreactive – B and T cells (72). ALPS patients often present with enlarged lymph nodes, spleen, and liver and a different spectrum of autoantibodies to that found in SLE (78). *PRKCD* deficiency is characterized by enhanced B cell proliferation and survival and impaired negative selection of self-reactive B cells (77).

RASopathies, like Noonan syndrome, are another group of diseases linked to lymphocyte apoptosis dysregulation. Clinical manifestations include distinctive facial features, short stature, heart issues, developmental delays, and occasionally, SLE-like disease (79). These conditions can be caused by variants in genes encoding RAS/MAPK pathway signaling proteins, including PTPN11, RAF, KRAS, NRAS, and SHOC2.

In addition, variants in genes that regulate lymphocyte development, activation, selection, and migration can also cause complex syndromes presenting with both immunodeficiency and autoimmunity that may present with lupus-like manifestations. GoF mutations in *PIK3CD* (29, 67, 80–82), that signals via AKT and mTOR to control the development and activation of T and B cells, result in a complex immunodeficiency syndrome referred to as activated PI3K δ syndrome, characterized by recurrent infections, lymphoproliferation, autoimmunity, and an increased risk of lymphoid malignancies (83). LoF variants of *P2RY8* associated with increased AKT and ERK activity, have also been found to impair B cell tolerance and found to be likely causes or contributors to SLE (84). B cells carrying pathogenic *P2RY8* variants could not be confined in germinal centers and negative selection of autoreactive B cells was impaired. DOCK8, a guanine nucleotide exchange factor predominantly expressed in lymphocytes, influences the actin cytoskeleton and lymphocyte migration and the activation of STAT3 (85). Hypomorphic variants in *SH2B3* that break B cell tolerance at least in part through increased IFN-γ and IL-4 signaling have also been recently reported to contribute to lupus-pathogenesis in humans and mice (86). Deficiency in *DOCK8* also leads to a combined immunodeficiency characterized by various features, including recurrent infections, autoimmunity and cancer (85), was associated with SLE in three case reports (87–89). Another case report (90) has also described a variation in *LRBA*, which regulates CTLA4 intracellular trafficking and protein expression (91) that was associated with a complex childhood-onset autoimmunity including colitis, type 1 diabetes and SLE. *BACH2* is a crucial transcription gene regulating T and B cell development, and its deficiency has been linked to SLE in mouse studies (92). Recently, a case of early-onset SLE in a BRIDA (BACH2-related immunodeficiency and autoimmunity) patient has suggested that *BACH2* variants may constitute a potential monogenic cause of SLE (93).

#### Metabolic defects

Defects in several metabolic pathways have sporadically been associated with monogenic lupus-like phenotypes. For example, LoF variants in *CYBB*, which encodes NOX2, a component of NADPH oxidase, typically cause chronic granulomatous disease (CGD) and a proportion of patients develop SLE (94). Defects in *SLC7A7* (encoding a subunit of a cationic amino acid transporter) can lead to lysinuric protein intolerance (LPI) and SLE (95). Deficiency in *PEPD* (encoding peptidase D, also known as prolidase, which regulates the recycling of proline) can result in SLE in a fraction of patients (96). Variants in *MAN2B1* (encoding lysosomal alpha-d-mannosidase) can cause lysosomal *α*-mannosidosis and have been found in four patients with SLE (97–99), one of which also carried an *SLC7A7* variant (99). If proven causative, these genetic alterations reveal a complex relationship between metabolic disruptions and autoimmunity that require mechanistic understanding.

### Implications for diagnosis and treatment

The increasing use of genomic sequencing in healthcare is assisting us in identifying an increasing number of potentially disease-causing genetic variations. The value of these findings depends on our ability to discern whether they are merely associated with disease or truly disease causing. Definitive genomic diagnosis holds significant implications for healthcare providers, patients, and their families as it can lead to a medical diagnosis, ending the diagnostic odyssey, provide families with severe hereditary diseases reproductive options, and guide the use of precision therapies. To aid in the interpretation of genetic findings, the National Institutes of Health established the ClinGen consortium in 2013 (100) with the objective of developing a reliable public resource that refines and establishes standards for evaluating the likelihood of a gene, and specific variants in each gene, being disease-causing and thus diagnostic. In 2022, a working group focusing on rheumatological autoimmune diseases was established, and in 2023 this group includes a gene curation expert panel (GCEP) with experts from multiple institutions that specifically focus on lupus (4). This effort is expected to lead to a refinement in the diagnosis and treatment of SLE, particularly early-onset forms.

A list of validated SLE-causing genes by the GCEP panel over the next two years will inform panel gene analysis of whole exome sequencing indicated for individuals with particularly severe or early onset SLE. Rare coding variants in validated lupus-causing genes can be classified according to ACMG guidelines (101). For those variants of unknown significance that do not reach the “likely pathogenic” or “pathogenic” classification criteria, assays that meet ClinGen guidelines for variant functional evaluation (102) will inform variant classification. There is a pressing need to develop such functional assays to test lupus variants, in ways that meet clinical and quality assurance standards.

While glucocorticoids, hydroxychloroquine, and immunosuppressive drugs continue to be the backbone of anti-inflammatory protocols in managing monogenic lupus, there is an increasing need for more targeted therapies to increase treatment efficacy. In some patients, lupus may be managed effectively with B cell-targeted treatments like Rituximab (103) and Belimumab (104), monoclonal antibodies that target CD20 and BAFF respectively. Recent studies, albeit in small numbers of patients, suggest that anti-CD19 chimeric antigen receptor (CAR) T cell therapy may outperform belimumab or rituximab, possibly through its ability to eliminate a greater number of tissue-resident B cells (105). An important role of type I IFN in disease progression suggests that medications designed to inhibit IFN-α/β receptors such as anifrolumab (106), may offer significant benefits to those suffering from mendelian SLE-like interferonopathies. Additional therapeutic options to decrease type I IFN signaling and formation of pathogenic ABCs cells include JAK inhibitors such as baricitinib and ruxolitinib (107, 108). Additionally, reverse transcriptase inhibitors, originally developed for HIV patients, have the potential to target endogenous retroviruses propose to be important SLE triggers (109) and reduce the IFN signature (110). Regular infusions of fresh frozen plasma (FFP) (111) and allogeneic hematopoietic stem cell transplantation (HSCT) may also prove to be effective options for patients with inherited complement deficiency or associated immunodeficiency.

Besides the generic treatment options described above, the question remains of how identification of functional rare variants in lupus genes may inform choice of therapy. For monogenic cases in which the variants occur within a gene confirmed to be lupus-causing or within genes clearly linked to others that are lupus causing, personalized treatment options may be possible. For example, patients identified to carry GoF *TLR7* variants (45), or LoF *UNC93B1* variants (52, 53) that disrupt degradative sorting of TLR7, may benefit from TLR7 inhibitors currently in phase II clinical trials. Additionally, mouse models of TLR7-driven lupus are dependent on IRAK4 (112), and several IRAK4 inhibitors are currently in phase I/II clinical trials (113), thus treatment with IRAK4 inhibitors may also be successful. Patients with pathogenic variants in specific DNases may benefit from administration of recombinant human DNase1 and DNase1L3 therapies. Dual-active DNases have recently been engineered and shown to be effective and degradation of NETs (114). Patients with *TREX1* loss of function variants that lead to overactivation of the cGAS pathway may benefit from small molecule cGAS inhibitors. Given the reported cross-talks between these pathways (109), it is not unreasonable to speculate that a particular precision therapy may be effective across several pathways.

A greater challenge will arise in non-mendelian lupus cases. It is however possible that a significant fraction of lupus cases may be oligogenic in nature: two or a small number of rare variants with strong effects may be responsible for a large component of disease susceptibility, just as is being proposed for heart disease, hypercholesterolemia, and neurodegenerative disease (115–117). These cases may benefit from combination therapies. A big unknown in the field is how many pathways are there to lupus, and whether many of the pathways today thought to be distinct interact or converge in one or a small number. At the rapid pace the field is moving, it is likely that interactions between some or most pathways described in this review will be revealed in the coming years.

## Conclusion

SLE is a complex autoimmune disease, the understanding of which has experienced significant advancements in the last few years, particularly through whole exome/genome sequencing using next-generation sequencing technologies. Over 30 genes have been recognized as causes of monogenic SLE or SLE-like disease. This information is providing improved mechanistic insights that promise to aid in the identification of effective biomarkers and therapeutic strategies.

## Figures and Tables

**Table 1:** Genes causing monogenic lupus.

Pathway	Gene (protein)^a^	MIM Associated disease	Number of reports of monogenic lupus (published year) (ref)^b^
Complement activation	*C1QA/C1QB/C1/QC*	C1Q deficiency	>30
	*C1R/C1S*	Ehlers-Danlos syndrome type 1;C1S deficiency	7
	*C2*	C2 deficiency	>20
	*C4A/C4B*	C4a and C4b deficiencies	>20
	*C3*	C3 deficiency	7
	*FCN3*	Immunodeficiency due to ficolin3 deficiency	2 (in 2019, 2020) (12, 13)
Activation of type 1 IFN pathway	*DNASE1*	SLE	1 (in 2001) (28)
	*DNASE1L3*	SLE and HUV	10
	*DNASE2*	Autoinflammatory-pancytopenia syndrome	3 (in 2020, 2021, 2023) (29–31)
	*TREX1*	AGS, FCL, SSd, SS	16
	*RNASEH2A/B/C* (RNASEH2)	AGS	1 (in 2015) (37)
	*SAMHD1*	AGS, FCL, CLL (somatic)	5 (in 2011, 2018, 2021, 2022) (32–36)
	*CECR1* (ADA2)	DADA2	2 (in 2016, 2017) (42, 43)
	*SAT1*	SLE	1 (in 2022) (44)
	*DDX58* (RIG–I)	SMS	1 (in 2023) (49)
	*TMEM173* (STING)	SAVI, FCL	2 (in 2014, 2017) (47, 48)
	*IFIH1* (MDA5)	AGS, FCL, SMS	1 (in 2015) (46)
	*TLR7*	SLE	1 (in 2022) (45)
	*UNC93B1*	HSE	2 (in 2024) (52,53)
	*IKZF1* (IKAROS)	CVID, ITP	2 (in 2017, 2019) (61, 62)
	*TNFAIP3* (A20)	Autoinflammatory syndrome, Behçet-like	3 (in 2019, 2020, 2021) (65–67)
	*ACP5* (TRAP)	SPENCD	8
	*PACSIN1*	NA	1 (in 2023) (56)
	*NLRC4*	Familial cold autoinflammatory syndrome, Autoinflammation with infantile enterocolitis	1 (in 2023) (58)
	*ISG15*	Immunodeficiency	1 (in 2021) (70)
T cell and B cell tolerance	*FAS/FASLG*	ALPS	1 (in 1996) (71)
	*PRKCD* (PKC delta)	ALPS	5 (in 2013, 2015, 2017, 2018, 2022) (72–76)
	*PTPN11/RAF/K RAS/NRAS/SHO C2*	Noonan syndrome	9
	*PIK3CD* (PI3K p110 δ)	Immunodeficiency	5 (in 2019, 2020, 2021, 2023) (29, 66, 79–81)
	*DOCK8*	Hyper–IgE syndrome, autosomal recessive, with recurrent infections	3 (in 2014, 2021) (85–87)
	*SH2B3*	Erythrocytosis, Myelofibrosis, Thrombocythemia	1 (in 2024)(86)
	*P2RY8*	NA	1 (in 2022) (83)
	*BACH2*	Immunodeficiency and autoimmunity	1 (in 2023) (90, 91)
	*LRBA*	Immunodeficiency, common variable, with autoimmunity	1 (in 2020) (88)
metabolism	*CYBB* (NOX2)	CGD	7
	*SLC7A7*	LPI	6
	*PEPD*	Prolidase deficiency	7
	*MAN2B1*	α-Mannosidosis, types I and II	3 (in 2004, 2019, 2022) (95–97)

HUV, hypocomplementaemic urticarial vasculitis; SSd, Sjögren syndrome; SS, systemic sclerosis; CLL, chronic lymphocytic leukaemia; HSE, herpes simplex encephalitis; ITP, immune thrombocytopenic purpura; NA, not available.

a Protein name specified in instances where it differs from the gene name.

b If the number of reports on gene variants leading to monogenic lupus is less than or equal to 5, the published year and references will be listed.

## Key points

- Monogenic lupus, caused by variants in single genes, is a rare severe form of SLE usually manifesting during childhood.
- Advancements in next-generation sequencing technologies have enabled the discovery of novel and ultra-rare variants causing monogenic SLE.
- Although monogenic lupus constitutes a small fraction of lupus cases, the discovery of its causative variants greatly enhances understanding of disease pathogenesis illuminating potential therapeutic strategies.
- Central to SLE pathogenesis are breakdown in nucleic acid tolerance, activation of the type I IFN pathway, and complement activation. Most gene mutations associated with monogenic lupus present with defects in these central mechanisms.
